# A Case of Adalimumab-associated Pancytopenia and Reversible Lymphadenopathy

**DOI:** 10.7759/cureus.3477

**Published:** 2018-10-22

**Authors:** Leonard T Walsh, Ravi Patel, Justin Loloi, Kirk Jones, Julie Caler, Zain Ayaz, Rohit Jain

**Affiliations:** 1 Internal Medicine, Penn State Milton S. Hershey Medical Center, Hershey, USA; 2 Pharmacology, Western Maryland Health System, Maryland, USA; 3 Internal Medicine, Western Maryland Health System, Maryland, USA; 4 Internal Medicine, Penn State Health Milton S. Hershey Medical Center, Hershey, USA

**Keywords:** adalimumab, pancytopenia, lymphadenopathy, ulcerative colitis

## Abstract

Adalimumab is a recombinant monoclonal antibody to tumor necrosis factor alpha (TNFα) used in the treatment of inflammatory and autoimmune conditions, including ulcerative colitis (UC). Adverse side effects include infection and injection-site cutaneous reactions; however, rare adverse events such as pancytopenia have been recorded. Here we describe a case of pancytopenia and reversible lymphadenopathy related to adalimumab administration in a patient with UC.

## Introduction

Adalimumab (Humira^TM^) is a subcutaneous self-administered recombinant fully-human monoclonal antibody to tumor necrosis factor alpha (TNFα), which is approved for use in patients with ulcerative colitis (UC). The drug binds with high affinity and specificity to soluble TNFα and neutralizes its biologic function by blocking its interaction with TNF receptors. Given the role of TNF in the inflammatory cascade, adalimumab has a central role in treating immune-mediated and inflammatory conditions, including rheumatoid arthritis (RA), psoriatic arthritis, ankylosing spondylitis, and inflammatory bowel disease.

The adverse effects of TNFα inhibitor therapy have been cited in studies beyond its use in UC. In a multicenter study, the BIOGEAS (Spanish Study of Biological Agents in Autoimmune Diseases) project, 379 cases of autoimmune diseases including cutaneous vasculitis, lupus-like syndrome/systemic lupus erythematosus (SLE), psoriasis, and interstitial lung diseases have been identified in association with the use of anti-TNF agents [1]. A meta-analysis of infliximab and adalimumab treatment in RA demonstrated that there is a dose-independent two-fold increased risk of serious infections and a dose-dependent risk of malignancy. Malignancy developed in 0.9% of patients treated with a TNFα inhibitor compared with 0.2% of patients given placebo [2]. Other rarely reported adverse effects included new onset or exacerbation of congestive heart failure and development of cytopenias [3].

There is a paucity of cases demonstrating the harmful effects of adalimumab on hematologic dyscrasias and lymphadenopathy. We present the case of a 70-year-old female with a history of UC who experienced pancytopenia and reversible lymphadenopathy as an adverse effect of adalimumab administration.

## Case presentation

**Figure 1:**
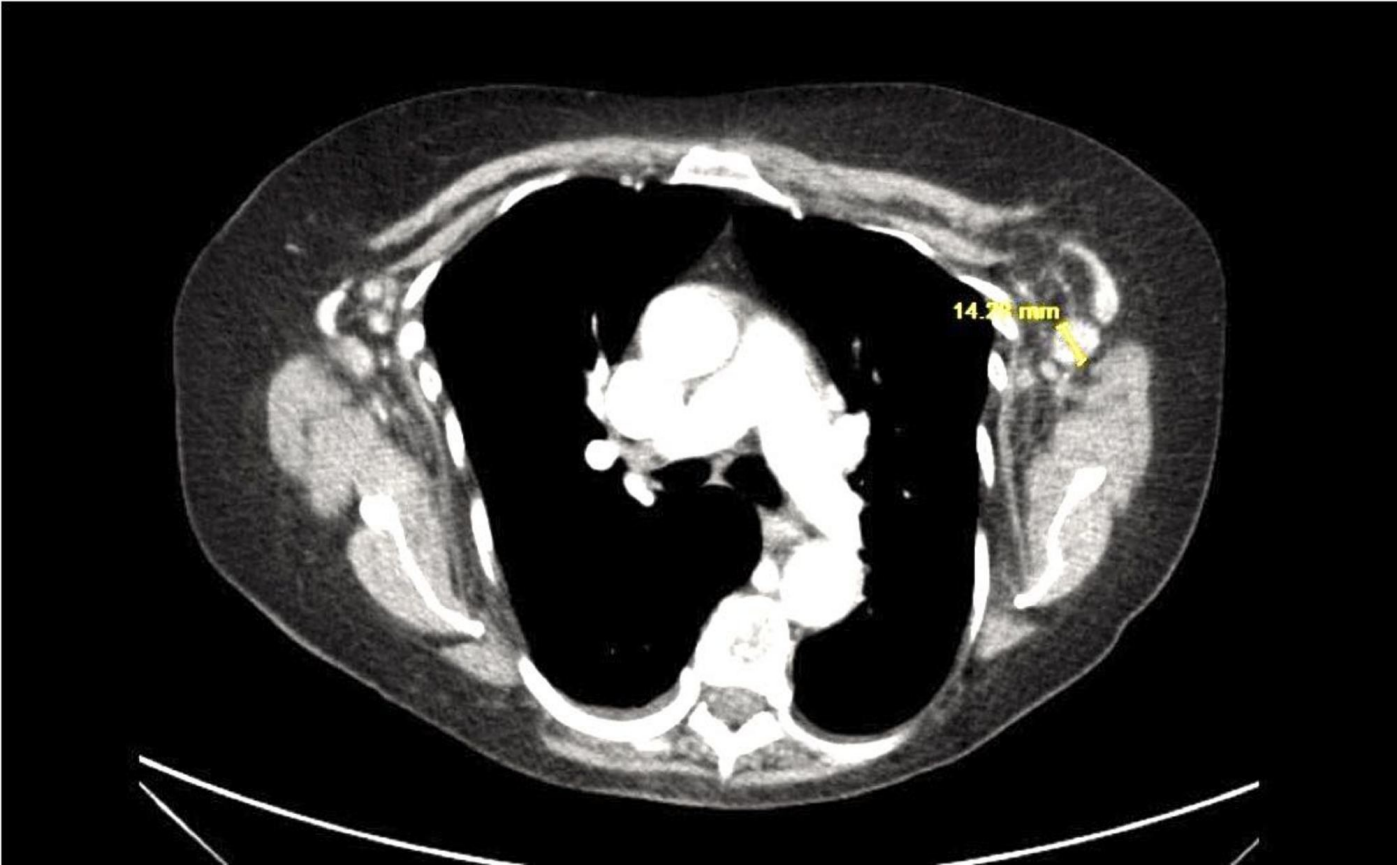
Chest CT showing bilateral axillary lymphadenopathy and enlarged retroperitoneal lymph nodes CT: computed tomography

**Figure 2:**
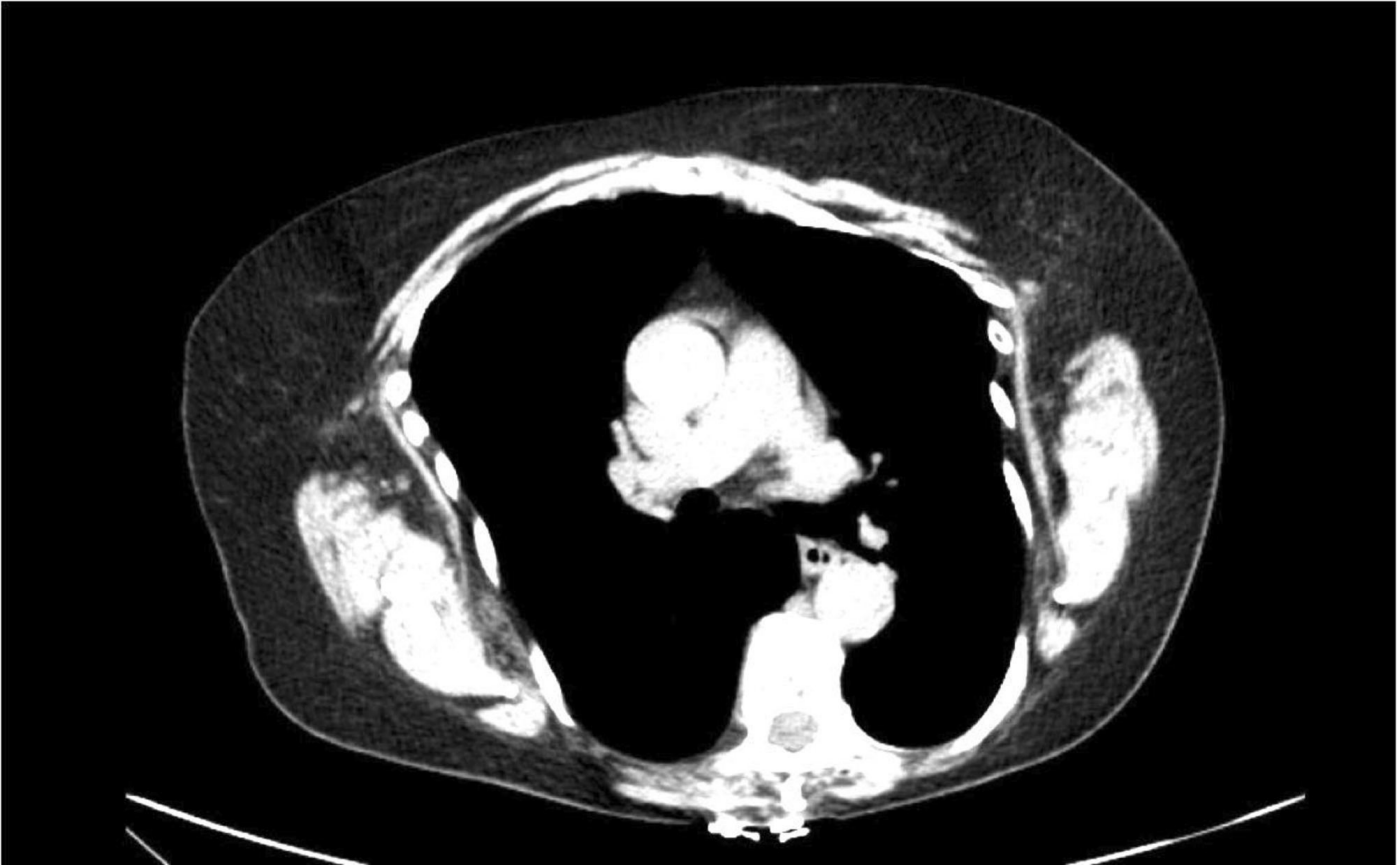
Two-week follow-up chest CT demonstrating resolution of lymphadenopathy CT: computed tomography

A 70-year-old female with a history of UC presented with a fever of 101℉ in the setting of progressive weakness over the last six months. Her UC was being treated with adalimumab, with her first dose at 15 months prior to presentation and the most recent dose one week prior to presentation. Physical exam was significant for predominant left submandibular lymphadenopathy without any rashes, bruising, or joint swelling. Labs showed pancytopenia with white blood cell (WBC) count 2.0 K/μL, hemoglobin 11.5 g/dL, platelets 10 K/μL, and absolute neutrophil count (ANC) 1.1 K/μL. A computed tomography (CT) scan of the chest, abdomen, and pelvis illustrated bilateral axillary lymphadenopathy (Figure 1) and enlarged retroperitoneal lymph nodes from the level of the celiac axis to the groin. Adalimumab was discontinued, infectious workup was initiated, and antibiotics were started for neutropenic fever. Shortly after, with the resolution of fever and negative infectious workup, antibiotics were discontinued. Tbo-filgrastim was administered with an increase in the WBC count to 8.2 K/μL and ANC to 6.2 K/μL. A lymph node biopsy revealed an atypical lymphoid proliferation with necrotizing granulomas. The patient remained clinically stable with a concordant increase in all cell lines in the following days and was discharged. A repeat CT scan performed two weeks later (Figure 2) illustrated a complete resolution of lymphadenopathy.

## Discussion

Anti-TNFα therapies carry many potential adverse infectious and non-infectious consequences, including hematological dyscrasias and lymphoma [4]. A multicenter prospective study, InspirADA, evaluated the effects of adalimumab on patients with moderate to severe UC. Treatment-emergent adverse events among 463 patients included infections (30%), allergic reactions (3.5%), malignancies (0.6%), worsening/new-onset psoriasis (0.6%), pancytopenia (5.4%), and injection-site reactions (9.9%) [5].

This case highlights an uncommon potential side effect of adalimumab, which has become a mainstay of treatment in patients with moderate-to-severe UC. In regard to diffuse lymphadenopathy, lymphoma continues to be a debated association. Long-term safety data from global clinical trials have recently illustrated that the malignancy rates for adalimumab-treated patients are as expected for the general population [6].^ ^With respect to cases of cytopenias, TNF inhibitor therapy is associated with a significant reduction in the peripheral blood neutrophil count [7].

Currently, there are no recommendations for screening or monitoring of hematologic dyscrasias for those receiving adalimumab. It is prudent for monitoring to be standardized for prescribing clinicians, as subclinical abnormalities can be intervened on with the discontinuation of therapy prior to the presentation of sequelae requiring hospitalization.

## Conclusions

This case highlights two rare complications, pancytopenia and lymphadenopathy, which have been attributed to adalimumab use. Despite this agent having recently gained mass appeal in the treatment of a wide array of inflammatory and immune-mediated conditions, there are no current recommendations for the screening and monitoring of hematologic abnormalities in those receiving adalimumab. We recommend that the prescribing physician actively monitor the status of a patient initiating adalimumab, as more rare and serious side effects such as pancytopenia can develop.
